# Editorial: The physiological effects of vibration therapy in health and disease

**DOI:** 10.3389/fphys.2024.1381145

**Published:** 2024-02-27

**Authors:** Danubia Cunha Sá-Caputo, Redha Taiar, Francois Constant Boyer, Amandine Rapin, Mario Bernardo-Filho

**Affiliations:** ^1^ Laboratório de Vibrações Mecânicas e Práticas Integrativas, Departamento de Biofísica e Biometria, Instituto de Biologia Roberto Alcantara Gomes and Policlínica Universitária Piquet Carneiro, Universidade do Estado do Rio de Janeiro, Rio de Janeiro, Brazil; ^2^ MATIM, Université de Reims Champagne-Ardenne, Reims, France; ^3^ Faculté de Médecine, Université de Reims Champagne Ardennes, Reims, France

**Keywords:** whole-body vibration, mechanical vibration, systemic vibratory therapy, acute effect, cumulative effect

## Introduction

When mechanical vibration is transmitted to the individual, whole-body vibration exercise (WBVE) is produced. WBVE can be used as a therapy (systemic vibratory therapy—SVT) or as a training intervention. Various effects of the WBVE have been described and contribute to improvements in muscular strength, bone mineral density, cognition, quality of life, and functional abilities, as well as to the reduction of the level of pain and risk of falls. SVT can be associated with other clinical procedures aiming to enhance the biological answers of the mechanical vibration in an acute or cumulative intervention (van Heuvelen et al., 2021; Sá-Caputo et al., 2023).

In this Research Topic, the readers will find a systematic review and meta-analysis about the effects of WBVE alone on physical function, activities of daily living, and quality of life in patients with stroke. The other articles are clinical studies about the effects of WBV 1) combined with maximal isometric voluntary contractions (MVCs) and steady isometric to evaluate the effects in the sensorimotor integration, 2) on energy expenditure during deep squats of male well-trained students, and 3) combined with KAATSU training on limb joint muscle strength.

The efficacy of WBVE in stroke patients was evaluated in a systematic review and meta-analysis Zeng et al. It was verified that variable frequency vibration and side-alternating vibration exhibited efficacy in reducing spasticity and improving motor and balance functions, while fixed frequency vibration and vertical vibration did not yield significant therapeutic benefits in these domains. It was concluded that WBVE may serve as a viable adjunct therapy for stroke patients to alleviate spasticity and enhance motor and balance functions.

Considering the effects of WBVE on performance have been related to potential changes in the neural drive, motor unit firing rate, and sensorimotor integration, motor unit coherence analysis was performed to detect the source of neural modulation based on the frequency domain Lecce et al. Men with maximal voluntary force (MVF) performed sustained contractions of the tibialis anterior (TA) muscle at 10%MVF before and after acute WBVE. High-density surface electromyography was used to record the myoelectrical activity of the TA to evaluate coherence from motor unit cumulative spike trains. Mean coherence showed a significant decrease in the alpha and low-beta bandwidths, whereas no significant changes were found in the other ones. According to the effects found in the alpha and low-beta bandwidths, which reflect sensorimotor integration parameters, accompanied by no differences in the discharge rate and the force covariance, the results underlined possible neural mechanisms at the base of the previously reported performance and that the mechanical vibrations that affect sensorimotor integration enhancements following acute WBVE are likely based on sensorimotor integration rather than direct neural drive modulation.

The effects of WBVE on the energy metabolism of deep squats with different weights were evaluated Huang et al. College students with sports experiences performed resistance exercise vibration (REV) or resistance exercise (RE) with varying loads two times per week for 4 weeks. It was found that the oxygen uptake and energy expenditure of the REV group were higher than those of the RE group during and 30 min after exercise, respectively, and the excess postexercise oxygen consumption (EPOC) was also higher than that of the RE group. Moreover, changes in the oxygen uptake and energy expenditure were stable with increasing exercise in both vibration and non-vibration conditions. It was suggested the WBVE can increase energy expenditure during low-intensity training and excess post-exercise oxygen consumption and improve exercise intensity.

KAATSU training (KT), also known as blood flow restriction training, causes muscle ischemia in the distal limb through compression. Moreover, KT stimulates muscle growth and improves muscle fitness at a relatively low exercise intensity (Golubev et al., 2020; Ishizaka et al., 2022). The effect of WBVE combined with KT on lower limb joint muscle strength was evaluated in older women Xiong and Liu. Healthy older people were divided into the WBVE group (WG), KT group (KG), combined intervention group (CIG), and control group (CG). WG and CIG subjects underwent WBVE, and KG and CIG subjects underwent 150 mmHg and lower limb joint and local compression intervention. The peak torque (PT) and endurance ratio (ER) of the joint flexion or extension were evaluated. The comparison among the groups at 16 weeks showed relative changes that were relatively significantly lower in the WG, KG, and CG compared to the CIG in the knee extension and ankle flexion PT. The relative changes were significantly greater in the WG, KG, and CIG compared to the CG in the knee extension PT. The relative changes were significantly lower in the WG, KG, and CG compared to the CIG in the ankle extension PT and the hip extension ER. The relative changes were significantly lower in the CG compared to the CIG in the knee extension ER. It was concluded that WBVE and KT increased the knee extensor strength in older women.

## Conclusion

The Research Topic “*The Physiological Effects of Vibration Therapy in Health and Disease*” presents important contributions of WBVE on health promotion. Putting together all the findings presented in this Research Topic about the physiological effects of vibration therapy in health and disease, it is possible to verify that WBVE alone or combined with other interventions can produce relevant biological answers in different populations. These effects will permit individuals to undergo improvements in their physical health and quality of life.
